# Gut Microbiota in Multiple Sclerosis and Experimental Autoimmune Encephalomyelitis: Current Applications and Future Perspectives

**DOI:** 10.1155/2018/8168717

**Published:** 2018-04-02

**Authors:** Fengna Chu, Mingchao Shi, Yue Lang, Donghui Shen, Tao Jin, Jie Zhu, Li Cui

**Affiliations:** ^1^Department of Neurology and Neuroscience Center, The First Hospital of Jilin University, Changchun, Jilin Province, China; ^2^Department of Neurobiology, Care Sciences & Society, Division of Neurodegeneration, Karolinska Institutet and Karolinska University Hospital Huddinge, Stockholm, Sweden

## Abstract

The gut environment and gut microbiome dysbiosis have been demonstrated to significantly influence a range of disorders in humans, including obesity, diabetes, rheumatoid arthritis, and multiple sclerosis (MS). MS is an autoimmune disease affecting the central nervous system (CNS). The etiology of MS is not clear, and it should involve both genetic and extrinsic factors. The extrinsic factors responsible for predisposition to MS remain elusive. Recent studies on MS and its animal model, experimental autoimmune encephalomyelitis (EAE), have found that gastrointestinal microbiota may play an important role in the pathogenesis of MS/EAE. Thus, gut microbiome adjustment may be a future direction of treatment in MS. In this review, we discuss the characteristics of the gut microbiota, the connection between the brain and the gut, and the changes in gut microbiota in MS/EAE, and we explore the possibility of applying microbiota therapies in patients with MS.

## 1. Introduction

Multiple sclerosis (MS) is an autoimmune central nervous system (CNS) disease, and experimental autoimmune encephalomyelitis (EAE) is an animal model of MS. The etiology and pathogenesis of MS and EAE remain unknown. Recently, studies have focused on the microbes that colonize the skin and mucosal surfaces and mainly those that may be found in the gastrointestinal (GI) tract [1–3]. It has been shown that an imbalance in the gut microbiota, called “dysbiosis,” is associated with various diseases, ranging from intestinal diseases, like colorectal cancer, irritable bowel syndrome, and inflammatory bowel disease (IBD) [4], to other systemic diseases such as obesity [5], malnutrition [6], diabetes, metabolic syndrome, and rheumatoid arthritis (RA) [7, 8]. In the field of neurology, attention is also focused on the role of the gut microbiota in CNS diseases, such as Alzheimer's disease and Parkinson's disease (PD) [9, 10]. Many recent studies have also found that the gut microbiota plays an important role in MS; this will help us find a new way to treat MS or prevent MS relapse.

## 2. The Microbiome

Human normal microorganisms consist of bacteria, microeukaryotes, fungi, yeast, archaea, helminths, protozoa, parasites, and viruses/phages, which are mainly distributed in the internal cavity of the body, such as respiratory tract, digestive tract, urogenital tract, and body surface, forming four microecosystems, with more than 95% of them located in the large intestine [11, 12]. All microbes including bacteria, archaea, fungi, and viruses exist in an ecosystem/habitat called microbiota, and the collective genomic, protein, or metabolite content of all the microbes in a given ecosystem/habitat called microbiome, for example, the microbial community, in the gut is called gut microbiota or gut flora [13]. There are about 10^14^ different populations of microorganisms in the human intestinal tract, which are at least 100 times larger than the number of human genes in the body, and their total weight is approximately 2 kg [14, 15]. Such a large number of intestinal microbes and hosts have evolved over a long period of time and have become an inseparable part of the host and play an important role in maintaining the body healthy. Because of the different physiological state of the human gastrointestinal tract, there are certain differences in the species and distribution of the bacteria, and even in the intestinal and intestinal mucus layers, the microbes is different. The exact species of microbe populations has not yet been determined. However, in recent years, the Metagenomics of the Human Intestinal Tract and the Human Microbiome Project have provided the most integrated view of human-associated microbes, and Hugon et al. list 2172 species isolated in humans, which they classified into 12 different phyla, with 93.5% of them belonging to the Proteobacteria, Firmicutes, Actinobacteria, and Bacteroidetes phyla [16, 17], and a healthy gut contains large fractions of the phyla Firmicutes and Bacteroidetes, including the genera *Prevotella*, *Bacteroides*, and *Ruminococcus*, followed by Verrucomicrobia and Actinobacteria but contains a low number of Proteobacteria phyla members [18]. The colonization of the human gut begins at birth and becomes relatively stable in adulthood. Different ethnicities should have different microbiomes, and the human gut microbiota does not remain constant, as it is affected by numerous factors [19].

### 2.1. Formation of Gut Microbiota and Influencing Factors

After birth, microbes rapidly colonize the sterile neonatal GI tract, and the microbiota composition partially depends on the delivery mode and whether breast-feeding is done [20–22]. It has been hypothesized that the different components of the gut microbiota after birth may have an impact on disease in the future [23]; research has found that a caesarean delivery and formula-feeding are associated with high incidences of infection and allergy diseases [24, 25]. One to 2.5 years after birth, the composition, diversity, and function of the infant microbiome gradually develops to resemble that of adults [26, 27]; in adulthood, most of the bacteria in the body will remain in a relatively stable state, but to the end of the life, the diversity of flora composition will decline and the ratio of Bacteroidetes/Firmicutes will increases; however, the human microbiota displays a remarkable degree of variation within and between individuals [28].

Recent evidence has shown that the adult microbiome is not as stable as previously believed, and there are many important endogenous and exogenous factors for the composition of the intestinal microbial community: (1) Genetic factors: There is an obvious adaptive relationship between host gene composition and bacterial gene composition. The bacteria can synthesize metabolites on the basis of genetic composition. These metabolites interact with each other, maintaining a stable balance between the gut microbiota and the surrounding environment. Even when twins and mother-daughter pairs had lived apart for many years, but they still have more similar microbiota compositions, suggesting that the gut microbiota may be influenced by genetics factors [29–31]. (2) Sex: The gut microbiota and sex hormone have been reported to influence each other, and research has found that males exhibited increased abundance of *Bacteroides* and *Prevotella* compared with females [32], and sex differences in the microbiome parallel immune, metabolic changes, it is important in risk and resilience of the disease throughout the lifespan [33, 34]. New studies in mice have found that adoptive transferred male microbiota to recipient females can result in elevated testosterone and metabolomic changes and delay the onset and lessen the severity of disease, which demonstrated that the female-biased risk for autoimmune disorders is significantly impacted by sex differences in the gut microbiome [35, 36]. (3) Diet: Dietary habits and food types can influence microbial composition [37–40]. (4) Drugs: Antibiotic and other drugs can easily affect the components of the microbiome [41, 42]. (5) Others: Other implicated factors include lifestyle, illness, smoking, drug addiction, place of residence, and the climate [43, 44].

### 2.2. Normal Function of the Gut Microbiota

An intimate mutualistic relationship between the gut microbiota and the host has been developed following thousands of years, and the gut microbes have become either harmless or beneficial to the host, maintaining the balance of the systemic and local immune systems. The human gut microbiota can synthesize and secrete essential vitamins to support immune regulation, endothelial growth, and the development of the CNS. Lactic acid bacteria can produce vitamin B12 that cannot be synthesized by other animals, fungi, or plants; *Bifidobacteria* are the main producers for folate, which is involved in DNA synthesis and DNA repair; other vitamins, including vitamin K, nicotinic acid, biotin, riboflavin, pantothenic acid, pyridoxine, and thiamine can also be synthesized by the human gut microbiota [45–48].

The beneficial effects of the gut microbiota on host metabolism are often considered to be mediated by short-chain fatty acids (SCFAs). These SCFAs can be absorbed in the GI tract by epithelial cells and be involved in the regulation of cellular processes like cytotaxis, proliferation, differentiation, and apoptosis [49].

Normal flora can systemically and locally stimulate the development of innate and adaptive immune systems and is required for normal immune system maturation, including gut-associated lymphoid tissue (GALT) development.

The enteric nervous system (ENS) is one of the main divisions of the autonomic nervous system and regulates the functions of the gastrointestinal tract, which has been described as a “second brain” [50]. ENS controls the motility, exocrine and endocrine secretions, and microcirculation of the gastrointestinal tract; it is also involved in regulating immune and inflammatory processes [51, 52].

### 2.3. Connection between Brain and Gut

**Figure 1:**
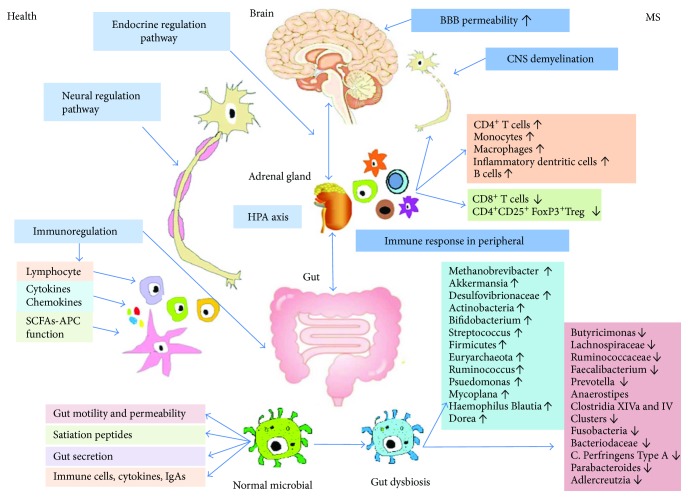
The role of the gut microbiota in health and MS. The gut microbiota can affect the body's nervous system function in numerous ways, including the neural regulating pathway, the endocrine pathway-HPA axis, and the immunoregulating pathway (via lymphocyte, cytokines, chemokines, and antigens presenting effect of SCFAs). The normal microbiome has many functions: (1) maintenance of the motility and permeability of the gut; (2) synthesis and secretion of essential vitamins, such as vitamin B12, folate, vitamin K, nicotinic acid, biotin, riboflavin, pyridoxine, panthotenic acid, and thiamine; (3) maintenance of intestinal epithelial functions, such as absorption and secretion; and (4) local stimulation of the development of innate and adaptive immune systems via GALT secreting immune cells, cytokines, and IgAs. When the gut microbiota is in dysbiosis, several diseases may develop, such as MS. The pathology of MS includes increased BBB permeability, destruction of the myelin layer in the CNS, and inflammatory cell infiltration of perivascular tissues. The immunological changes of MS in the periphery are characterized by an increase in pro-inflammatory effector such as CD4^+^ T cells, monocytes, macrophages, inflammatory dendritic cells, and B cells and a decrease in CD8^+^ T cells and CD4^+^ CD25^+^ FoxP3^+^ Treg cells [84, 87–90]. Patients with MS can exhibit gut microbial dysbiosis, with increases in *Methanobrevibacter* Akkermansia [128], Desulfovibrionaceae [132], Actinobacteria, *Bifidobacterium*, *Streptococcus* [137], Firmicutes, Euryarchaeota [133], *Ruminococcus* [138], Pseudomonas, Mycoplana, *Haemophilus*, Blautia, and Dorea [140] and decreases in Butyricimonas [128], Lachnospiraceae, Ruminococcaceae [132], Faecalibacterium, *Prevotella*, Anaerostipes, Clostridia XIVa and IV Clusters [137], Fusobacteria [133], Bacteroidaceae [138], *C. perfringens* type A [139], Parabacteroides, and Adlercreutzia [140]. BBB: blood-brain barrier; CNS: central nervous system; HPA axis: hypothalamic-pituitary-adrenal axis; SCFA: short chain fatty acids; APC: antigen presenting cell; GLAT: gut-associated lymphoid tissue; FoxP3: forkhead box 3; Treg: regulatory T cells.

At the end of the 19th century, the American scientist Gershon first described the concept of gut-brain connection [50]. Over the past decade, a large number of animal and preclinical studies have proven that the gut microbiota is involved in regulating physiological processes in humans, such as host metabolism and immunity, and can modulate brain signals, triggering bidirectional signaling via the microbiome-gut-brain axis through the endocrine, immune, nervous, and metabolic systems [53, 54]. This axis includes a variety of molecular pathways interacting with each other (Figure 1).

#### 2.3.1. Neural Regulation Pathway

The gut microbiota can secrete and regulate neurotransmitters of the central and peripheral nervous systems; intestinal lymphocytes can be stimulated by local environmental changes in the lumen (including changes in the gut microbiota) and release cytokines to activate the endocrine or paracrine systems and consequently affect the CNS. At the same time, the CNS can directly impact the gut via sympathetic nervous system or parasympathetic nervous system, especially of the vagus nerve; the regulations are mainly mediated by the secretion of catecholamines or acetylcholine, which influence ENS circuits [55, 56]. Gut microbes also produce a range of important components that are implicated in neuroactive and immune regulation, including secreting *γ*-aminobutyric acid (GABA), histamine, serotonin, dopamine, and others [57, 58].

#### 2.3.2. Endocrine Regulation Pathway

The hypothalamic-pituitary-adrenal (HPA) axis comprises the hypothalamus and the pituitary and adrenal glands. When confronted with stress or other stimulants, the HPA axis finally releases glucocorticoids, mineralocorticoids, or catecholamines; all of them can alter gut microbiota composition and increase gut epithelium permeability and immune responses [59–61]. Increased corticosterone levels in stressed mice lead to intestinal dysbiosis that is characterized by the *Clostridium* genus increase and the *Bacteroides* genus decrease [62]. Because glucocorticoids have both proinflammatory and anti-inflammatory effects on the peripheral and CNS immune cells, and inflammatory and autoimmune diseases are often associated with impaired HPA axis functionality, such as in RA, IBD, and MS [61, 63, 64].

#### 2.3.3. Immunoregulation Pathway

Gut microbes can modulate the immune response in a variety of ways, such as affecting antigen presenting effect and regulating the production of cytokines and the function of lymphocytes. The gut microbiota plays an important role in the fermentation of indigestible carbohydrates into the three most abundant SCFAs: acetate, propionate, and butyrate. In the human gut, acetate is produced by gut anaerobes, propionate is significantly produced by *Bacteroidetes*, and butyrate production is mainly by Firmicutes [65–67]. These molecules activate the brain's immune response, which can trigger inflammation in the nervous system and cause a series of neurological symptoms. Butyrate has anti-inflammatory and anticancer functions, and it constitutes an important energy source for colonocytes and has an effect in inhibiting histone deacetylase (epigenetics) [68], generating intestinal and circulating regulatory T (Treg) cells [69], maintaining blood-brain barrier (BBB) integrity [70], and modulating CNS-microglia activity [71]. SCFAs have also been known to have strong anti-inflammatory effects. They can influence the production of cytokines and have relationship with the G-protein-coupled receptor 43 (GPR43) to elicit an anti-inflammatory effect [72]. The GI microbiome can regulate the development of the host innate and adaptive immune systems via the gut–CNS-axis. The microbes are necessary for host immunity generation because in the GI tract, they can format GALTs. The GALT represents the largest immune holder in the human body, containing nearly 80% of the immune compartments. The regulatory T cells and autoimmune pathogenic T cells may maturate in the GALT and suppress autoimmune response outside the gut [15, 73]. Studies with germ-free (GF) mice have shown a thinner mucus layer and Peyer's patches and decrease numbers of secreting IgA plasma cells, CD4^+^ T cells, and antimicrobial peptides [74–76]. In GF mice, the lymph nodes and spleens are abnormally developed, with decreased numbers of B and T cells in the germinal centers and parafollicular region [77].

#### 2.3.4. Metabolic System Regulation Pathway

Gut microbes modulate brain function by the release of metabolites such as immune antigens (peptidoglycan, lipopolysaccharide (LPS), and polysaccharide A (PSA)) with immunological effects [78, 79]. Normal bacteria can stimulate the production of cross-reactive antibodies (mainly IgA). These antibodies are secreted into the intestine and can play an important role in preventing bacterial infection. PSA derived from *Bacteroides fragilis* is known as an immunomodulator with inhibitory function in CD39^+^ FoxP3^+^ T cells and Treg cells [80]. Lipid 654, produced by *Bacteroidetes* in the human gut, can be a ligand for mouse TLR2 and human and exist in the systemic circulation of healthy humans [81] (Figure 1).

## 3. MS/EAE and Gut Microbes

### 3.1. MS and EAE

MS is an autoimmune disease in the CNS, which is the main cause of disability in young people in Western countries [82, 83]. Pathological changes associated with MS include the loss of BBB integrity, inflammatory cell infiltration of perivascular tissues, destruction the myelin layer, and axonal damage [84]. The clinical features may be diverse and include limb weakness, paresthesia, fatigue, blurred vision, and cognitive deficits, among others [85]. EAE is the most widely used animal model of MS and resembles its pathological, clinical, and immunological features [86]. The immunological changes in MS/EAE are characterized by increasing proinflammatory cell infiltration, followed by CD4^+^ T cells with the Th1 or Th17 phenotypes, monocytes, macrophages, inflammatory dendritic cells, and B cells, and decreasing in CD8^+^ T cells, CD4^+^ CD25^+^ Forkhead box 3 (FoxP3^+^) Treg cells, and impaired Treg function [84, 87–90].

The etiology of MS remains relatively unknown, and it may include both genetic and environmental factors. Genetic factors: MS often occurs in young women, and the ratio of female to male in MS has increased to an incidence of 3 : 1 over the past decades, indicating a potential role of hormones in the occurrence of MS. Androgens have the ability to reduce the natural killer (NK) cells, toll-like receptor 4, and tumor necrosis factor-alpha (TNF-*α*), while they upregulate anti-inflammatory molecule production, such as interleukin-10 (IL-10). In contrast, estrogens may enhance the production of the proinflammatory cytokines like IL-1, IL-6, and TNF-*α* [34, 91]. Over 100 genetic risk factors have been identified for MS, including HLA alleles (HLA-DRB1^∗^1501, DR4, and DR3), transcription factors, adhesion molecules, chemokines, cytokines, and micro-RNA genes [92]. However, the MS is not fully controlled by genetics, as monozygotic twins, sharing 100% of genetic material, show an approximately 25–30% lifetime risk for MS when one of them has been diagnosed, suggesting that the genetic background could be interacting with other risk factors [93, 94]. Environmental factors: Previously, it was reported that living at higher latitudes would pose a higher disease risk because of more limited sun exposure leading to possible vitamin D deficiency [95]. Other environment factors include smoking, antibiotic exposure, vaccination, obesity, ethanol abuse, EB virus infection, exposure to air pollutants including PMs (particulate matters), heavy metals, and airborne biological pollutants such as lipopolysaccharide (LPS) and gut microbiota changes [96–101]. Recently, it was found that many of the risk factors listed, including reduced vitamin D intake, smoking, hypercaloric Western diet, vaccination, stress, and alcohol addiction, may be related to the gut microbiome dysbiosis [102]. A reduced vitamin D intake can alter the immune responses, producing FoxP3^+^ Treg cells and reducing T cells in the gut, which could affect gut microbial populations directly [103]. Smoking can influence the gut microbiome in humans; after smoking cessation, microbial diversity increases and the overall composition of the microbiome changes [104]. Therefore, the dysbiosis of gut microbiota may be involved in the pathogenesis of MS.

### 3.2. The Microbiome in EAE

The impact of the gut microbiota on the development of MS has been rooted in several preclinical studies in EAE. The gut microbiota plays an essential role in the occurrence and development of the immune system in EAE; it can regulate BBB permeability, limit astrocyte pathogenicity, activate microglia, and express myelin genes [70, 71, 105, 106] (Table 1).

Germ-free (GF) mice show significant defects in both systemic lymphoid and gut-associated tissues, and Peyer's patches are hypoplastic in such animals with greatly decreased number of plasma cells, which produce resident CD4^+^ T cells and IgA [107]. Although EAE can be induced in GF mice, the severity of EAE was obviously reduced [108, 109], due to the inability to form more pathogenic T cells such as Th17 cells. However, when these mice are colonized with segmented filamentous bacteria, they show a recirculate inflammation in EAE and enhanced disease severity, which increases Th17 cells in the CNS [108]. The gut microbiota may be necessary in the normal BBB development, since germ-free mice have disrupted BBB tight junction and increased BBB permeability compared to controls [70].

Because the gut microbiota has immune regulation functions, attempts have been made to treat EAE by inducing changes in the gut microbiota. Ochoa reported that treatment with oral antibiotics in EAE can reduce intestinal symbiotic gut and delay the development of EAE, while the intraperitoneal injection of antibiotics in mice had no obvious impact on the development of EAE, which suggests that gut microbiota changes are associated with the development of EAE [42]. The protective effect of antibiotic treatment in EAE is related with a regulation of the abnormal in T cell responses in the CNS and the GI tract, diminishing proinflammatory cells, like Th1 and Th17 cells and their cytokines IFN and IL-17A and enhancing anti-inflammatory response, including increasing secretion of FoxP3^+^ Treg cells, IL-10 and IL-13 [108–112]. Except for changes in T-cell subsets, B cells can recruit and dendritic cells can activate Th1 and Th17 cells in EAE [108, 109]. Some studies have suggested that some bacterial strains have beneficial effects on EAE by protecting mice from disease exacerbation [113–116]. One of the common bacteria *B. fragilis* has the ability to produce polysaccharide A (PSA), which can induce naive T-cell differentiation to produce IL-10 FoxP3^+^ CD4 Treg cells and protect mice from CNS demyelinating diseases [114, 117]. The oral administration of PSA could have both preventive effect and therapeutic effect to protect against EAE [102]. Other bacteria such as the *Bifidobacterium* may also decrease EAE symptoms [113]. The oral administration of *Lactobacillus* spp. and other lactic acid-producing bacteria have been demonstrated to reduce the clinical score of EAE [111, 118, 119]. Mangalam recently showed that *Prevotella histicola* can suppress disease in EAE, as it induced CD4^+^ FoxP3^+^ regulatory T cells and tolerogenic dendritic cells and suppressed macrophages [13, 120]. CD44 deficiency alters three phyla (Bacteroidetes, Firmicutes, and Proteobacteria) of gut microbes, which in turn may play a crucial role in suppressing inflammatory T-cell differentiation accounting for the amelioration of EAE [121]. Autoreactive CD4^+^-induced intraepithelial lymphocytes, which are influenced by stimuli from the gut environment, can also suppress activity against T cell-mediated EAE [73]. The oral administration of *Salmonella typhimurium* could increase Treg frequency and decrease Th1 and Th17 cells, which would lead to a decrease in EAE clinical score [115, 122, 123]. Additionally, recent studies have indicated that *Bacteroides fragilis* and Clostridia clusters XIVa and IV, which derived from human feces, may have the ability to induce Foxp3^+^ Treg and be able to suppress inflammatory response in EAE [114, 124, 125].

### 3.3. Gut Microbiota in MS

As mentioned above, the gut bacteria have a symbiotic relationship with the host, which could help the host maintain a healthy stable state. At the phylum level, the fecal microbiota is mainly constituted of Bacteroidetes and Firmicutes and with smaller amounts of Verrucomicrobia, Euryarchaeota, and Proteobacteria. In the last few years, several studies have demonstrated that patients with MS exhibit gut microbial dysbiosis with both enrichment and depletion of certain bacterial populations compared to healthy controls (Table 2). Recently, the studies reported that fecal content isolated from the patients with MS transferred to mice increased EAE incidence or severity, which provide the evidence that MS-derived microbiota contain factors that regulate adaptive autoimmune responses and precipitate an MS-like autoimmune disease in a transgenic mouse model, suggesting the potential functional effects associated with altered microbiotas observed in MS and targeting microbiota as a therapeutic strategy in MS [126, 127].

Jangi et al. showed increases in *Methanobrevibacter* (Euryarchaeota phylum) and in *Akkermansia* (Verrucomicrobia phylum) and decreases in *Butyricimonas* in MS, which correlates with the gene expression of interferon signaling, dendritic cell maturation, and NF-kB signaling pathways in circulating monocytes and T cells [128]. In treated patients with MS with interferon-*β* (IFN-*β*) and glatiramer acetate (GA), there was an increased number of *Sutterella* and *Prevotell*a and a decreased number of *Sarcina* compared to those in untreated patients [128]. *Akkermansia* have immunoregulatory effects on changing mucin into SCFA, and they could also play a reverse role in degrading the mucus layer in proinflammation function [129, 130]. The *Butyricimonas* species are butyrate-producing bacteria and have anti-inflammatory action by inducing Treg cells in the gut, and a decrease in *Butyricimonas* will decrease SCFA production [131]. Tremlett conducted three experiments in pediatric MS and found that Firmicutes, Archaea Euryarchaeota, and Proteobacteria (Desulfovibrionaceae) increase in patients with MS, while Lachnospira (Lachnospiraceae), Verrucomicrobia (Ruminococcaceae), and Fusobacteria decrease, and MS in children with the absence (versus presence) of Fusobacteria was associated with relapse risk [132–134]. Tremlett found no difference in immune markers between MS and controls; however, she discovered that Bacteroidetes were inversely associated with Th17 only in MS and Fusobacteria correlated with Tregs only in controls [134]. A study with 20 patients with MS and 40 healthy controls in Japan demonstrated that altered intestinal microbiota in patients with relapsing remitting MS (RRMS) involved increased *Actinobacteria*, *Bifidobacterium*, and *Streptococcus* and decreased *Bacteroides*, *Faecalibacterium*, *Prevotella*, *Anaerostipes*, and Clostridia XIVa and IV clusters. Clostridium clusters XIVa and IV constitute a 10–40% of the bacteria in the healthy gut [135–137]. The MS patients with expanded disability status score (EDSS) ≤ 3.0 who received GA treatment were shown to have larger numbers of *Bacteroidaceae, Ruminococcus, Lactobacillaceae*, and *Clostridium* compared to the numbers seen in untreated patients [138]. After vitamin D supplementation, *Faecalibacterium* increases in GA naive MS relative to GA-treated MS and healthy controls [138]. Rumah et al. isolated epsilon toxin- (ETX-) producing *Clostridium perfringens* type B from a young woman with MS and found that 10% of their patients with MS had ETX-specific antibodies compared to only 1% of controls. ETX can disrupt the BBB and bind with myelin, which may be a potential trigger of MS [139]. In contrast, *Clostridium perfringens* type A which is commensal with humans was nearly 50% in healthy controls and only 23% in MS patients [139]. Chen et al. also reported dysbiosis in 31 patients and 36 controls in a study with patients with RRMS and increased abundance of *Pseudomonas, Mycoplana, Haemophilus, Blautia*, and *Dorea genera* and depleted *Parabacteroides, Adlercreutzia*, and *Prevotella* in patients with MS [140]. Dorea have been considered to be part of the healthy gut microbiota, but its higher abundance in patients with MS and IBD has suggested a proinflammatory role for this bacterium, and Schirmer et al. showed that Doreacerat in species can induce IFN-*γ*, metabolize sialic acids, and degrade mucin for its proinflammatory functions [13, 141, 142]. Apart from the gut, one study also reported the presence of bacteria in brain biopsies. Biopsy samples of brain white matter showed a higher abundance of Actinobacteria in RRMS and Proteobacteria in primary progressive MS (PPMS) and a decrease of Actinobacteria in PPMS [143]. Recently, Farrokhi et al. demonstrated unique lipopeptide bacteria that originate from serine lipopeptide, lipid 654, which is produced by some Bacteroidetes commensal species, providing further evidence for an association between the bacteria and MS. Kleinewietfeld et al. demonstrated that lipid 654 is expressing at lower levels in the MS patients' serum than in healthy controls'. So lipid 654 may be a useful biomarker to evaluate MS activity [144, 145].

Overall, patients with MS usually have gut dysbiosis and often reduced numbers of *Faecalibacterium*, Bacteroidaceae, and *Prevotella*. After drug therapy, the gut microbiota of patients with MS changes; thus, regulating gut bacteria could a future direction for treatment in MS.

## 4. Treatments in MS

Currently, the primary goal of therapy in MS is symptom improvement after a disease attack, preventing new attacks and decreasing the rate of neurodegeneration in the CNS. Existing therapies available for patients mainly rely on nonspecific treatments, such as corticosteroids, immunosuppressants, and immunomodulating drugs, which often result in drug resistance or severe side effects [146]. As mentioned above, the gut microbiota plays an important role in the development of MS. Alterations in the gut microbiota in MS/EAE can also influence the clinical symptoms and inflammatory factors, which could help us find a new strategy or target to treat MS.

### 4.1. Dietary Modification

As mentioned earlier, dietary habits can affect intestinal microbe composition. There is an obvious difference in the gut microbiota composition between obese and normal-weight individuals, and obese individuals have reduced diversity in their microbiome especially at a lower level of Bacteroidetes [30]. Studies on the effect of Westernized diet with high fat on mice have shown changes in the gut flora, with an increasing in proinflammatory plasma free fatty acids and increased severity in EAE [147, 148]. The gut microbiota in the mice can be changed easily within only one day when their diet switches from a plant polysaccharide-rich, low-fat diet to a high-sugar/high-fat Western diet, which also changes microbiome metabolic pathways and alters the gene expression of the microbiome [102]. A restricted calorie diet can improve the EAE symptoms, whereas a high-salt diet causes disease exacerbation in EAE by promoting the expansion of macrophages and proinflammatory T cells, and Th17 differentiation, and also causing restraint in remyelination [113, 118, 149–152]. Middle- and long-chain fatty acids from dietary intake or microbial production promote pathogenic T-cell differentiation in the gut and then induce CNS inflammation. Conversely, SCFA can lead to disease amelioration by protective regulatory T-cell expansion [153]. A study also showed a similar trends on MS in human, suggesting that high sodium intake would worsen the disease [154]. Vitamin D levels are changed in the gut microbiome in MS, which can promote the differentiation of Treg, and the level of Vitamin D is important in maintaining microbiome system balance [103, 155, 156]. Studies have recently showed a direct connection between dietary tryptophan and the symptoms of EAE; deficiency of the antihyaluronidase reaction in astrocytes or lack of dietary tryptophan will fail to recover during the chronic stage of EAE [92].

### 4.2. Drugs

Antibiotic drugs can easily affect the components of the microbiome. Numerous studies have found that oral antibiotics can reduce in intestinal symbiotic gut and delay the development of EAE [42, 110, 114, 157, 158]. Broad-spectrum antibiotics can alter the population of T cells in the GALT and in peripheral lymphoid tissues to reduce the susceptibility to EAE, and the total number of Foxp3^+^ Tregs was significantly increased with a corresponding increase in IL-10 production [159]. A study that administered ciprofloxacin in healthy volunteers over a 10-month period found that the fecal microbiota reached a stable state similar, yet distinct, from the pretreatment state [160]. Minocycline is a kind of tetracycline antibiotic that often used for treating acne, which was also used to reduce disease severity in EAE both prophylactically and therapeutically [137]. Antibiotic therapy may be beneficial in the treatment of MS.

Other drugs, such as fingolimod, teriflunomide, and dimethyl fumarate, have immunomodulatory functions and have been shown to inhibit *C. perfringens* growth; therefore, the inhibition of *C. perfringens* may contribute to the clinical efficacy of these disease-modifying drugs [161].

### 4.3. Probiotics Treatment

Studies have suggested that probiotics can influence systemic immune responses, and the mechanisms behind the efficacy of probiotics may include maintaining the function of the gastrointestinal–epithelial barrier, increasing antimicrobial peptide production, and helping the activation of the host immune system in response to pathogens. Thus, probiotics could be used as adjuvant therapy to treat immune-mediated diseases [162, 163].

In recent years, an increasing number of animal experiments have provided evidence that the administration of probiotics can improve CNS symptoms. *Long bifidobacterium* (b. Longum), *Breve bifidobacterium*, *Bifidobacterium infantis*, *Lactobacillus helveticus*, *Rhamnose lactobacillus*, plant *Lactobacillus*, and *Lactobacillus casei* have been shown to effectively improve behavior, such as anxiety and depression, in animal models [164].

Infection with *Lactobacillus casei* Shirota or oral *Lactobacillus farciminis*, *Bifidobacterium bifidum*, *Bacteroides fragilis*, and *Bifidobacterium animalis* in mice resulted in Treg cell induction by promoting the secretion of IL-10, which was followed by IFN-*γ*, TNF-*α*, and IL-17 reduction and inflammatory Th1/Th17 decrease, and were shown to be EAE resistant or reduced the symptoms of MS [111, 113, 114, 118, 149, 165, 166].

Takata et al. found that *Candida kefyr* (*C. kefyr*) could alleviate the severity EAE symptoms. The bacteria can reduce the quantity of intestinal lamina propria Thl7 cells and cause IL-6 decline; at the same time, Tregs in the mesenteric lymph nodes and CDl03^+^ regulatory dendritic cells increase. The analysis of 16s-rRNA in rats showed the increased incidence of *Lactobacillus* in the feces and decrease in polymorphic *Bacillus* [167].

The periodontal *Porphyromonas gingivalis* may enhance glial cell activation and proinflammatory responses and exacerbate EAE [168, 169]. In contrast, *Candida kefyr* found that fermented foods can reduce the EAE [167].

Recently, studies have shown that using heat-killed bacteria like probiotic *Pediococcu sacidilactici* strain R037, PSA purified from *B. fragilis* [165], and heat shock protein 65 (Hsp65) produced from the *Lactococcus lactis* [170] also reduced the severity of EAE [114, 115, 118, 167, 170]. These findings suggest that bacteria-derived products may have therapeutic potential in MS and EAE.

### 4.4. Fecal Microbial Transplantation (FMT)

Currently, FMT has been paid wide attention for restoring intestinal microecological balance, which may be significantly efficacious and have less adverse reactions. Now FMT's adaptive diseases has been extended from the initial intestinal disorders to the metabolism, neurosis, autoimmunity, allergic diseases, and cancer prevention [171–173]. The results of clinical trials have shown that FMT can improve the walking ability in MS and alleviate autistic behavior as well as improve the neurological symptoms of PD [171, 174]. Borody et al. reported three patients with MS with severe constipation treated with FMT, which reduced the neurological symptoms and normalized walking. Unfortunately, the study had a small sample and was uncontrolled [175]. So far, FMT has been limited to individual cases, and clinical applications require more rigorous scientific evidence and human experimental verification in large samples.

### 4.5. Others

Parasites, in particular helminths or worms, have an effect on Th2 cell induction to produce anti-inflammatory cytokines, including IL-4, IL-10, IL-13, and TGF-b [176]. Helminths have provided therapeutic effects in patients suffering from MS and ulcerative colitis [176]. In addition, patients with MS naturally infected with helminths had fewer relapses than uninfected patients, and elimination of the parasites worsened their condition [177, 178]. Based on these findings, helminths may be a new method of MS treatment.

As described above, the alterations in the microbial composition of the gut may drive disease, which is a process called as dysbiosis. In recent years, more and more evidence suggest that the dysbiosis of the gut microbiome may be the cause of MS and other disorders, such as depression and Parkinson's disease [179–181]. However, the pathogenesis of these disorders is more complex than the dysbiosis of gut microbiome speculated [182], which may be one of the pathogenic factors for driving these disorders. Also it might be an alteration of microbial architecture from healthy condition with immune balance shifting toward an immune imbalance with inflammatory phenotype [183].

## 5. Conclusion

This review focused on exploring the complex roles of the alterations in the gut microbiome in MS and EAE. Dysbiosis in the gut microbiome may be one of the causes of the numerous diseases, including MS. Gut therapies including dietary modification, drug treatment, probiotics, FTX, and perhaps helminth treatment may be used in MS in the future. However, there is still a long way to go, as more rigorous scientific evidence with larger sample sizes are required for clinical application.

## Figures and Tables

**Table 1:** Microbes and inflammatory factors in EAE.

Author	Intervention	Function	IF increase	IF decrease	Microbial changes
Lee et al. [108]	GF mice versus CC mice	Resistant to the development of EAE in GF mice	CD4^+^ CD25^+^ Foxp3^+^ Tregs	IFN-*γ* and IL-17A	
Berer et al. [109]	GF mice versus SPF RR mice	Reduce the percentage of EAE in GF mice	CD4^+^ T cells	TH17 T-cell receptor (TCRablow)	
Ochoa-Reparaz et al. [42]	Antibiotic (ampicillin, neomycin sulfate, metronidazole, vancomycin), minocycline	Protects mice against EAE; reduced the severity of EAE	IL-13, IL-10 FoxP3^+^ Treg cells CD4^+^ or CD8^+^ T cell	IFN-*γ*, MIP-1*α*, MIP-1*β*, MCP-1, IL-17, and IL-6	
Yokote et al. [110]	Antibiotics (KCV)	Suppressed the development of EAE		IFN-*γ*, TNF-*α*, IL-6, and IL-17 Th17 iNKT cells	Reduction of *Lactobacillus murinus* and *Bacteroides fragilis* and increase in *Bacteroides thetaiotaomicron*
Ochoa-Reparaz et al. [114]	(1) Antibiotics (2) Antibiotics + WT *B. fragilis* (3) Antibiotics + △PSA *B. fragilis* (4) Mice treated with PBS	(1) Antibiotics: reduce EAE severity; delays clinical onset. (2) Antibiotics + WT *B. fragilis*: protect against disease; reduced clinical severity	(1) Antibiotics: IL-13, GATA-3 (2) WT *B. fragilis*: IL-10, IFN-*γ*, IL-12, GATA-3, SMAD-3 (3) △PSA *B. fragilis*: ROR*γ*t, IL-17, and T-bet	(1) Antibiotics: T-bet, IFN-*γ*, IL-17 and IL-6 (2) WT B. fragilis: RORgt, IL-17 (3) △PSA B. fragilis: GATA-3, IL-10, and IL-13	In WT or △PSA *B. fragilis* group: increase *Bacteroides* spp. counts
Ochoa-Reparaz et al. [157]	(1) Antibiotic (ampicillin, metronidazole, vancomycin, and neomycin sulfate) (2) Adoptively transferred CD5^+^ B cells	Both groups can reduce the severity of EAE	Enhances the frequency of IL-10 producing CD1d^high^ CD5^+^ B cells		
Ochoa-Reparaz et al. [165]	Oral administration with purified PSA	Both prevention and therapeutic effect on EAE	CD103 expressing		
Wang et al. [116, 184]	Treatment with PSA versus PBS	Delayed clinical onset and progression of EAE	CD39^+^ CD4 T cells CD39^+^ Foxp3^+^ CD4 Tregs		
Jun et al. [122, 123]	Treatment with *Salmonella typhimurium*	Reduced clinical development and protection against EAE	IL-17, IL-4, IL-10, and IL-13 Th1 and Th17	TGF-*β*, IFN-*γ*, Foxp3^+^ CD4^+^ T cells	
Ochoa-Reparaz et al. [115]	Treatment with *Salmonella typhimurium*	Reduced clinical scores and reduced disease duration	CNS inflammatory cell infiltration; CD25^+^ CD4^+^ T cells FoxP3^+^ Treg cells		
Ezendam et al. [113]	*Bifidobacterium animalis*	Reduced the duration of clinical symptoms of EAE			
Ezendam and van Loveren [166]	*Lactobacillus casei* Shirota	Increased the duration of clinical symptoms of EAE			
Lavasani et al. [111]	Lactobacilli	Prevents and therapy of EAE	IL-4, IL-10 and TGF-*β*1 IL-27	TNF-*α*, IFN-*γ*	
Takata et al. [118]	*P. acidilactici*	Prevent and therapy of EAE	CD4^+^ IL-10 producing cells CD4^+^ FoxP3^+^ cells		
Maassen and Claassen [119]	Lactobacilli	Suppress the disease			
Kwon et al. [149]	Orally IRT5	Prevent and therapy of EAE	IL-2, IL-4, IL-10	Th1/Th17; IFN*γ*, TNF*α*, and IL17	
Rezende et al. [170]	*L. lactis*	Prevented the development of EAE	IL-10, CD4^+^ FoxP3^+^ Treg cells and CD4^+^ LAP^+^ Tregs	IL-17	
Chitrala et al. [121]	CD44 deletion and fecal transfer	Amelioration of EAE	Change in SCFAs: propionic acid and i-butyric acid		Dominant in Bacteroidetes phylum and low in Firmicutes phylum.
Scott et al. [41]	Omeprazole treatment	No difference in clinical scores			Increase unidentified bacteria in S24-7 and decrease in *Akkermansia muciniphila* and *Coprococcus* sp.
Mangalam et al. [120]	Administration of *Prevotella histicola*	Suppressed EAE induced	CD4^+^ FoxP3^+^ Tregs DCs, IL-10	IL-17 and IFN-*γ*	

IF: inflammatory factor; GF: germ-free; CC: conventionally colonized; EAE: experimental autoimmune encephalomyelitis; CD: cluster of differentiation; Foxp3: Forkhead box P3; Tregs: regulatory T cells; IFN-*γ*: interferon-*γ*; IL: interleukin; RR: relapsing–remitting; MIP: macrophage inflammatory protein; MCP: monocyte chemoattractant protein; KCV: kanamycin, colistin, and vancomycin; TNF-*α*: tumor necrosis factor-*α*; PBS: phosphate buffer solution; iNKT cell: invariant natural killer T cell; WT *B. fragilis*: wild type *B. fragilis*; △PSA *B. fragilis*: PSA-deficient *B. fragilis*; ROR*γ*: RAR-related orphan receptor gamma; T-bet: T-box transcription factor TBX21; DCs: dendritic cell; TGF-*β*: transform growth factor-*β*; PSA: polysaccharide A; IRT5: consisting of *Lactobacillus casei*, *Lactobacillus acidophilus*, *Lactobacillus reuteni*, *Bifidobacterium bifidum*, and *Streptococcus thermophiles*; LAP^+^: latency-associated peptide; Hsp: heat shock proteins.

**Table 2:** Microbes in MS.

Author	Subject	Treatment	Bacterial in MS↑	Bacterial in MS↓	Bacterial in treatment↑	Bacterial in treatment↓	Other results
Jangi et al. [128]	MS *n* = 60 HC *n* = 43	IFN-*β n* = 18 GA *n* = 14 Untreated *n* = 28	Methanobrevibacter Akkermansia	Butyricimonas	Prevotella Sutterella	Sarcina	
Tremlett et al. [134]	Children ≤ 18 MS *n* = 15 HC *n* = 9	IFN-*β n* = 2 GA *n* = 5 Untreated *n* = 8					(1) No difference in immune markers between MS and HC. (2) Associations between immune markers (Th17, Tregs) and gut microbiota (Bacteroidetes and Actinobacteria) had been noted.
Tremlett et al. [132]	Children ≤ 18 MS *n* = 18 HC *n* = 17	IFN-*β n* = 3 GA *n* = 5 Natalizumab *n* = 1 Corticosteroids *n* = 6 Untreated *n* = 9	Desulfovibrionaceae (Bilophila, Desulfovibrio, Christensenellaceae)	Lachnospiraceae Ruminococcaceae			(1) Observed children very early in their MS which close to MS onset. (2) Onset of MS with more subtle changes rather than in the community composition.
Branton et al. [143]	PPMS *n* = 5 RRMS *n* = 4 SPMS *n* = 14 HC *n* = 21		Actinobacteria in RRMS Proteobacteria in PPMS	Actinobacteria in PP-MS			(1) Using brain biopsies for MS, and RNA sequence analysis for bacteria. (2) Composition of the bacteria maybe different from gut.
Wilson et al. [137]	MS *n* = 20 HC *n* = 40		Actinobacteria Bifidobacterium Streptococcus	Bacteroides, Faecalibacterium, Prevotella, Anaerostipes Clostridia XIVa and IV Clusters			
Tremlett et al. [133]	MS *n* = 17	IFN-*β n* = 3 GA *n* = 5 Natalizumab *n* = 1 Untreated *n* = 8	Firmicutes Archaea Euryarchaeota	Fusobacteria			Absence (versus presence) of Fusobacteria was associated with relapse risk
Cantarel et al. [138]	MS *n* = 7 HC *n* = 8	GA *n* = 5 Untreated *n* = 2 Vitamin D	Ruminococcus	Faecalibacterium, Bacteroidaceae	Faecalibacterium		Faecalibacterium increased for GA naïve MS relative to GA-treated MS and HC.
Rumah et al. [139]	RRMS *n* = 26 SPMS *n* = 4 HC *n* = 31			*C. perfringens* type A			
Chen et al. [140]	MS *n* = 31 HC *n* = 36	IFN-*β n* = 14 GA *n* = 1 Natalizumab *n* = 5 Untreated *n* = 11	Pseudomonas, Mycoplana, Haemophilus, Blautia, Dorea	Parabacteroides, Adlercreutzia, Prevotella			
Rumah et al. [161]		Fingolimod DMF Teriflunomide				*C. perfringens*	Inhibition of *C. perfringens* growth may contribute to the clinical efficacy of MS

MS: multiple sclerosis; HC: health control; GA: glatiramer acetate; IFN-*β*: beta-interferon; PPMS: primary progressive multiple sclerosis; RRMS: relapsing remitting multiple sclerosis; SPMS: secondary progressive multiple sclerosis; DMF: dimethyl fumarate. ↑ means increase and ↓ means decrease.
